# Immunofluorescence analysis of cytokeratin 8/18 staining is a sensitive assay for the detection of cell apoptosis

**DOI:** 10.3892/ol.2015.2856

**Published:** 2015-01-07

**Authors:** QIAO-MEI DONG, CHUN LING, LI ZHAO

**Affiliations:** Central Laboratory, The First Hospital of Lanzhou University, Lanzhou, Gansu 730000, P.R. China

**Keywords:** 4′,6-diamidine-2′-phenylindole dihydrochloride, cell apoptosis, flow cytometry, immunofluorescence staining, cytokeratin 8/18

## Abstract

Apoptosis is one of the major types of programmed cell death. During this process, cells experience a series of morphological and biochemical changes. Flow cytometric analysis of Annexin V staining of cell surface phosphatidylserine, in combination with a DNA-staining dye to probe the permeability of the cell membrane, is an established method for detecting apoptosis. The present study aimed to show that the immunofluorescence analysis of cytokeratin (CK) 8/18 staining may provide a new and sensitive assay for the detection of apoptotic cells. Tumor cells were treated with 20 μM cisplatin to induce apoptosis. Following 12 and 24 h of cisplatin treatment, cells were collected and stained with 4′,6-diamidine-2′-phenylindole dihydrochloride (DAPI) and fluorescein-labeled anti-CK8/18 antibody. The apoptotic cells were subsequently examined by fluorescence microscopy. Annexin V-fluorescein isothiocyanate/propidium iodide staining followed by flow cytometric analysis confirmed that cisplatin was able to induce apoptosis in tumor cells. Immunofluorescence analysis demonstrated that apoptotic cells had a distinct CK8/18 staining pattern. In living cells, CK8/18 was uniformly distributed in the cytoplasm and cytosol; however in the apoptotic cells with a condensed and/or fragmented apoptotic nucleus (as identified by DAPI staining), fluorescein-labeled anti-CK8/18 antibody exhibited unusual punctate and/or bubbly staining in the cytosol. In the apoptotic cells that could not be identified by DAPI staining, fluorescein-labeled CK8/18 displayed polarized aggregated staining in the cytosol. These results indicate that fluorescein-conjugated CK8/18 may be a useful and sensitive indicator of cell apoptosis.

## Introduction

Cell apoptosis is a form of programmed cell death. Such cell death is actively regulated by mitochondria-related intrinsic signaling or death receptor-stimulated extrinsic signaling, which induces a cascade of caspase-mediated photolytic degradation of their substrates (1–2). Consequently, during the process of apoptosis, cells undergo a series of morphological and biochemical changes (3); these have formed the basis for a number of methods of apoptotic cell detection (4). For example, the majority of apoptotic cells exhibit distinct changes in nuclear morphology, including nuclear condensation and fragmentation, which may be easily detected by propidium iodide (PI) or 4′,6-diamidine-2′-phenylindole dihydrochloride (DAPI) staining (5–6). The egression of phosphatidylserine from within the cell membrane to the cell surface during apoptosis provides a biological marker for apoptotic cells; this can be detected by Annexin V staining. In addition, during apoptosis, the cytoskeleton undergoes a clear reorganization, which is considered to be an important characteristic of apoptotic cells (7–9). In the process of tumorigenesis, apoptosis is inhibited, therefore, the detection of apoptosis is important for evaluation the efficacy of chemotherapy drugs. Double staining with V-FITC/PI and DAPI are classic apoptosis detection methods, however, a more specific method is required. Few methods using fluorescent-labelled cytoskeletal components in apoptosis have been reported, therefore, in the present study, alterations in the cytoskeleton during apoptosis were examined using fluorescein-conjugated anti-cytokeratin (CK) 8/18 staining.

## Materials and methods

### Chemicals

Roswell Park Memorial Institute (RPMI) 1640 medium was purchased from Invitrogen (Carlsbad, CA, USA). Fetal bovine serum (FBS) was obtained from Hyclone (Logan, UT, USA). DAPI was obtained from Sigma-Aldrich (St. Louis, MO, USA). Cisplatin was purchased from Shandong Qilu Medical Biotechnology (Beijing, China). The Annexin-fluorescein isothiocyanate (FITC) apoptosis detection kit was purchased from Beijing Baosai Biotechnology (Beijing, China). Anti-ck8/18 antibody was purchased from Abcam (Cambridge, MA, USA, catalog no. ab32118)

### Cell culture and treatment

The human gastric cancer cell line SGC-7901 (Shanghai Institute of Biological Science, Chinese Academy of Sciences; Shanghai, China) was cultured in RPMI 1640 medium supplemented with 10% FBS, and incubated in an atmosphere of 100% humidity and 5% CO_2,_ at 37°C. For treatment with cisplatin, cells were plated in 6-well plates at a density of 1.5×10^5^ cells/well in 2 ml RPMI 1640. Following a 24 h incubation, the medium was replaced with medium containing cisplatin at a final concentration of 20 μM to induce apoptosis. Control cells were treated with cisplatin-free medium. Cells were incubated at 37°C for the indicated time (12 or 24 h) and subsequently collected by centrifugation for 5 min at 300 × g at 4°C for further analysis (10).

### Detection of apoptosis by flow cytometry

To evaluate apoptosis in the cells, an Annexin V-FITC apoptosis detection kit was used according to manufacturer’s instructions. Cells were collected by centrifugation for 5 min at 300 × g at 4°C and resuspended in 200 μl binding buffer (part of the Annexin V-FITC apoptosis detection kit; Beijing Baosai Biotechnology). Annexin V-FITC (10 μl) and PI (5 μl) were added to the suspension and incubated for 15 min at room temperature. A further 300 μl binding buffer was subsequently added, and apoptosis was detected using a flow cytometer (Beckman; Palo Alto, CA, USA) to identify Annexin V+ and or PI+ cells. Experiments were performed in triplicate. The apoptosis rate was calculated in accordance with the formula: (Number of early apoptosis cells + number of late apoptosis cells)/total cell number.

### Immunofluorescence and fluorescence microscopic imaging

Cells were placed on glass slides by cytospinning at 1,000 rpm for 10 min at 4°C and fixed with 4% paraformaldehyde, prior to permeabilization with 0.06% of triton X-100 and blocking in 2% bovine serum albumin. Following overnight incubation with fluorescein-conjugated anti-CK8/18 antibody at 4°C, the cell nuclei were counterstained with 10 μl DAPI (stock solution, 1:1 DAPI:glycerol) (11).

### Statistical analysis

All statistical comparisons were performed using SPSS software, version 16.0 (IBM, Armonk, NY, USA). The Student’s t-test was used to compare differences between the two groups or association. P<0.05 was considered to indicate a statistically significant difference.

## Results

### Cisplatin effectively induces apoptosis in SGC-7901 cells

**Figure 1:**
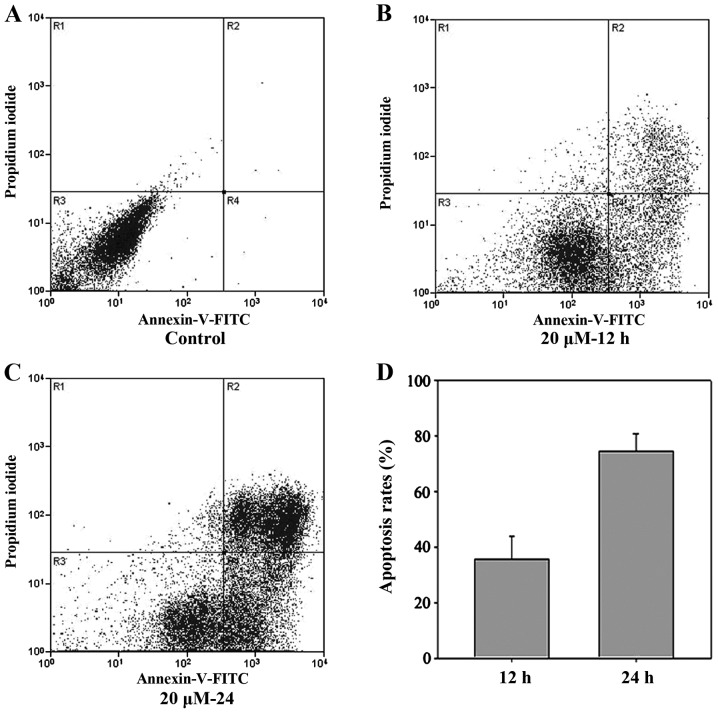
Cisplatin-induced apoptosis in SGC-7901 cells. Flow cytometric analysis of Annexin V/propidium iodide staining was used to analyze apoptosis in SGC-9701 cells following (A) vehicle-treatment (control), or treatment with 20 μM cisplatin for (B) 12 h and (C) 24 h. (D) The percentage of apoptotic cells was compared between cell populations treated with cisplatin for 12 or 24 h. FITC, fluorescein isothiocyanate.

The percentage of apoptotic SGC-7901 cells induced by cisplatin was evaluated by Annexin V and PI staining followed by flow cytometric analysis. The results indicated that cisplatin was able to effectively induce apoptosis in SGC-7901 cells and that the rate of apoptosis was time dependent. The rate of apoptosis at 12 h (36.37±3.11%) was lower than that at 24 h (75.87±4.11%) (P<0.01; Fig. 1); this difference was significant (P<0.05). Additionally, the difference between the control and cisplatin-treated cells was also significant (P<0.05).

### Fluorescein-conjugated anti-CK8/18 antibody staining revealed a unique cytoskeletal pattern in apoptotic cells

**Figure 2:**
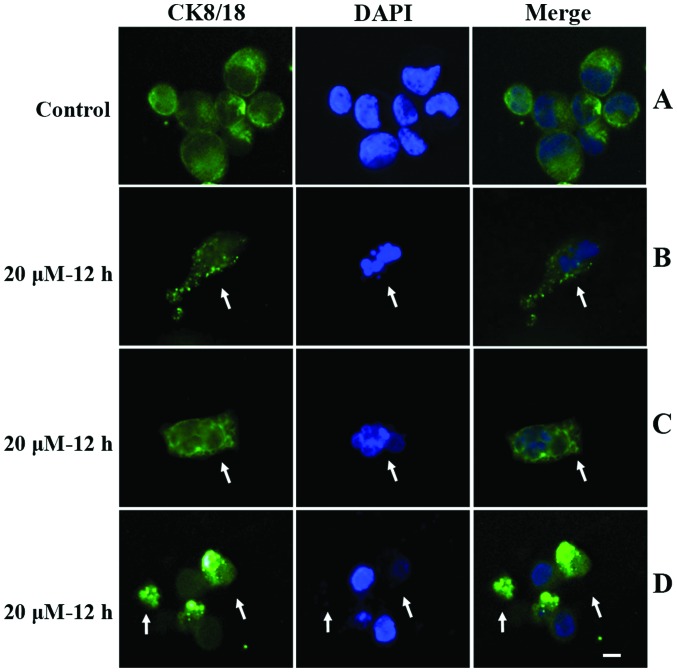
Fluorescein-labeled anti-CK8/18 antibody was used to detect apoptotic cells. (A) Control cells showed regular nuclear DAPI staining with uniform distribution of cytoplasmic CK8/18 staining. Apoptotic cells with fragmented nuclei exhibited (B) punctate or (C) bubbly CK8/18 distribution in cytosol. (D) Apoptotic cells with a split nucleus and weak DAPI staining also exhibited punctate or aggregated CK8/18 staining in the cytosol. Arrows indicate apoptotic cells. Scale bar, 10 μm. CK, cytokeratin; DAPI, 4′,6-diamidine-2′-phenylindole dihydrochloride.

To study the alterations in the cytoskeleton during apoptosis, SGC-7901 cells were collected following a 12 h incubation with 20 μM cisplatin. Cells were placed on slides by cytospinning and stained with fluorescein-conjugated anti-CK8/18 antibody (green) and DAPI (blue). The results are shown in Fig. 2 (arrows). In vehicle-treated control cells, very few apoptotic cells were detected (rate of apoptosis, 3.64±2.85%) (Fig. 2A), and CK8/18 was uniformly distributed in cytosol of almost all cells. However, in cisplatin treated cells, DAPI staining revealed a population of cells with a condensed and/or fragmented nucleus, indicating apoptosis. In these cells, punctate and/or bubbly CK8/18 staining was observed in the cytosol (Fig. 2B and C, arrow). Additionally, in cisplatin treated cells, a subset of late apoptotic cells with a split nucleus and weak nuclear DAPI staining also exhibited punctate, bubbly or aggregated cytosol distribution of fluorescein-conjugated anti-CK8/18 antibody (Fig. 2D, arrows).

## Discussion

CKs are major structural proteins of epithelial cells, which belong to the intermediate filaments family. They are highly conserved between species and are important for maintaining the integrity and continuity of epithelial cells. CKs comprise at least 20 members that are classified into two categories: The acidic type I group (CK9-CK20) and the neutral-basic type II group (CK1-CK8). Type I CKs are co-expressed with type II CKs to form heterodimers (12–13).

CK18 is a type I keratin, encoded by a gene located on chromosome 12q13. Its complementary type II keratin is CK8. In the absence of CK8, CK18 is degraded and keratin intermediate filaments are not formed (14). CK8 and 18 are co-expressed and serve as structural molecules that maintain cytoplasmic structure, resist external stresses, and participate in a number of cellular processes (14). These CKs are primarily expressed in adult epithelial organs, including the liver, lung, gastrointestinal tract and kidney, and also in cancer cells arising from these tissues (15–17). They are involved in the regulation of tumor metastasis, and have also been associated with progression and prognosis in various types of epithelial cancers. CK8/18 may therefore act as a diagnostic marker (18–19). In esophageal squamous cell carcinoma, CK8/18 expression is significantly increased in the advanced stages of the disease (20–21). Similar phenomena are observed in other malignant diseases, including gastric cancer and lung cancer (22). The present study used the gastric cancer cell line SGC-7901 as a model, in which high levels of cytosolic CK8/18 expression were observed, as indicated by immunofluorescence staining (Fig. 2A).

Apoptosis is one of the major types of programmed cell death. During this process, cells experience a series of morphological and biochemical changes, including membrane blebbing, cell shrinkage, cytoskeletal rearrangement, nuclear fragmentation, chromatin condensation, and chromosomal DNA fragmentation (23). The majority of methods used for the detection of apoptotic cells are based on these changes. Annexin V-FITC/PI-flow cytometry method is a standard detection method, and is capable of distinguishing early apoptotic, late apoptotic and necrotic cells (10,24). Annexin V is a calcium-dependent phospholipid binding protein. It binds to the membrane component, phosphatidylserine, which is redistributed to the extracellular surface of the cell membrane during apoptosis. PI is a nucleic acid dye. In the present study, Annexin V-FITC/PI-flow cytometry was used to confirm that 20 μM cisplatin was able to effectively induce apoptosis in SGC-7901 cells. DAPI staining revealed that nuclear changes occurred in a population of apoptotic cells.

Cytoskeletal rearrangement during apoptosis was investigated using a fluorescein-conjugated anti-CK8/18 antibody. Distinctive cytoskeletal patterning was observed in cells with specific apoptotic nuclear alterations and also in late apoptotic cells displaying a split nucleus and weak nuclear DAPI staining (Fig. 2B–D, arrows), distinct from that of living cells.

In conclusion, fluorescein-conjugated anti-CK8/18 antibody staining is a novel and effective technique for apoptotic cell detection, particularly in tissues with high levels of CK8/18 expression. Our findings indicate that immunofluorescence analysis of cytokeratin 8/18 staining is a sensitive assay for detecting cell apoptosis. As this study is preliminary, further research is required to confirm these results. In the future, studies investigating whether other pro-apoptotic chemicals may induce these types of changes would be extremely beneficial.
